# Timing Information Propagation in Interactive Networks

**DOI:** 10.1038/s41598-019-40801-5

**Published:** 2019-03-14

**Authors:** Imane Hafnaoui, Gabriela Nicolescu, Giovanni Beltrame

**Affiliations:** Computer and Software Engineering Department, Polytechnique Montreal, Montreal, QC H3T 1J4 Canada

## Abstract

Animal behavior is greatly influenced by interaction between peers as well as with the environment. Understanding the flow of information between individuals can help decipher their behavior. This applies to both the microscopic and macroscopic levels, from cellular communication to coordinated actions by humans. The aim of this work is to provide a simple but sufficient model of information propagation to learn from natural coordinated behavior, and apply this knowledge to engineered systems. We develop a probabilistic model to infer the information propagation in a network of communicating agents with different degrees of interaction affinity. Another focus of the work is estimating the time needed to reach an agreement between all agents. We experiment using swarms of robots to emulate the communication of biological and social media groups for which we are able to provide upper bounds for the time needed to reach a global consensus, as well as to identify individuals that are responsible for slow convergence.

## Introduction

Behaviors in a large group of individuals that change the state of a system are referred to as collective behaviors. Although the term was first used by sociologists^1,2^ to refer to the emergence of new social structures as a reaction to certain events, it was later extended to cover behaviors observed in the animal kingdom such as in schools of fish^3^, flocks of birds^4^, and ant colonies^5^. There is a general agreement among sociologists and biologists as to the conditions that encourage the emergence of collective behavior. The most prominent ones are conflict, ambiguous policies^6^, or change in the normative order^7,8^. The detection of a new food source, for instance, is observed to trigger behaviors ranging from establishing optimal routes by ants^5^ to nest migrations of bee swarms^9^. When an intruder is sensed, hyenas use unique whoops, specific to every individual, to reach a consensus on who belongs to the clan and then use the whoops to coordinate the hunt against the intruder^10^.

Studying these intricate systems has taken one of two main directions: a macroscopic view, which focuses on the group-level behavior, like the study of the group morphology^11,12^; or a microscopic view that aims at studying the interactions between individuals which give rise to the behaviors observed in aggregations^13^. Generally, a collective behavior does not emerge from the state of the individual entities in a group, whether that be emotions of uncertainty, imagery or strain in the natural order. It is rather the result of the information shared between the individuals in a communication network. A good example of this behavior is the spreading of rumors in social networks. A previous study^14^ showed that social network platforms are increasingly becoming the go-to media to share information among directly and indirectly affected individuals in case of a crisis. Even officials, such as emergency responders, are becoming reliant on these media to gather and communicate information^15^. As such, the study of how information spreads, rumors in this case, becomes necessary to stave off potential emergence of chaotic social behaviors. At a microscopic level, the brain can be likened to the systems mentioned so far in that neurons are equipped with neurotransmitters that propagate signals through a neural network to give rise to a given function. Similarly, scientists have recorded collective behaviors in cancer cells similar to those observed in animal groups in which patterns of collective alignment is observed to generate collective cell migrations, recognized to be at the crux of tumor invasions^16–18^. All of these systems can be abstracted and represented as networks of interacting agents (animals, users, cells, robots) propagating some kind of information (visual queues, pheromones, tweets, chemical signals, messages) for the purpose of changing the global behavior of the group.

Luckily, information can be quantified, its flow measured and its representation bounded. The limits as to the way information is described, processed or delivered is dictated by the physics of the system. One model to represent the communication in a network is the simple-to-define proximity network^19^. Here, the assumption is that individuals in close proximity interact with each other. However, in reality, the reception of information is hindered by various conditions such as a noisy environment, the affinity of an individual to cooperation, etc. On top of this, assuming that a dependence relationship exists only among the individuals in close proximity and is therefore at the epicentre of the the emergence of collective behaviors is rather naive. In an effort to move past this simple model, we introduce a stochastic element to the interactions between individuals in their range of communication. The next section defines the characteristics of this probabilistic variable and alludes at the physical elements in nature that can be modelled with it.

Our contribution is two-folds. First, we aim to probabilistically model the propagation of information in a network of interacting agents. Many works have proposed complex models to represent the propagation of information, especially infection spreading^20–23^ with a number of parameters and settings. These models, for instance, are built on the assumption that the infection rate, the state of the individual, time and age of infection, etc. are known which might actually be difficult to acquire in a real case-study. The work proposed in^21^ goes as far as to require a pedestrian model to accurately estimate the infection transmission in air travel. In addition, most proposed propagation models rely on scenario-specific parameters such as an Susceptible-Infected-Recovered (SIR) model in studying infection spreading which cannot be used to study the propagation of signals in animal groups for instance. The strength of our model lays in abstracting the quantity being propagated to a piece of information (visual queues, chemical signals, messages) with the likelihood of transmission as an attribute (line of sight, infection probability, influence of users). This eliminates the dependence of the model on the scenario being studied and renders it applicable to multiple domains of study. In here, we model the information propagation by stripping away these details down to a fewer number of assumptions; namely (1) a static or slowly changing network, (2) the propagation of a single piece of information and (3) information transmission probability of a node. The purpose here is to show that this simple model is enough to explain the emergence and occurrence of certain events. Behaviors like synchronized flashing exhibited by *photonius carolinus* fireflies and the tendency of certain fireflies to defect from the group is one interesting scenario to study^24^. In that regard, the flashing lights are construed as information being sent to the rest of the group. The model is then a tool to infer the influence of these defectors on others out of their line of sight by studying the propagation of information between individuals in different regions. As a sub-contribution, we explore the validity of centrality as a contributor to information propagation. Oftentimes, centrality has been a key parameter to techniques that dealt with detecting propagation sources and selecting influential nodes in the network^25–27^. In this work, we reveal the drawbacks of relying on centrality and propose a metric based on conviction and influence probability to boost the propagation of information.

Second, armed with this model, considering that one of the incentives to studying collective behavior in nature is to gather the knowledge to engineer new systems (e.g. optimized transportation routes, robot swarms as emergency responders), we analyse the timing characteristics of information propagation and the ways it could lead to new technologies. To illustrate this, we study leader-following consensus, common in decision-making problems within groups of interacting individuals, in which the purpose is for the group to reach and agree on the opinion of a leader. This can be modelled as an information propagation paradigm in which an opinion is an information for which the convergence means the agents in the network agreeing to that argument. This has been observed to be a feature that groups, both animal and human, strive for to make decisions and establish certainty over a choice of action^28^. When it comes to consensus, the research is focused on developing controllers that are more resilient and those that guarantee convergence. To our knowledge, little has been dedicated to explore the convergence times towards information propagation. The work of Başar *et al*.^29^ defines the expected convergence rate of Quantized Metropolis consensus. The assumption though is that the graph remains connected in every sequence and that the transmission occurs to a single node per step with a uniform probability. A noisy environment increases the chances of information loss, and inter-individual conflicts might arise, especially within heterogeneous groups. All this increases the time needed to reach a consensus. This has been considered by Cheng *et al*.^30^ in which the effect of noise-attenuation gain was explored in leader-following consensus to define a bound to the convergence rate. The results were limited to gains of a certain class of functions. From the field of evolutionary graph theory, the authors^31^ define the exact fixation probability and time of a Moran process for arbitrary small graphs. Most of these works rely on Markov chains to model the information propagation which renders the states intractable as the network grows in size. Our aim is to provide a probabilistic estimate of information propagation time which is of practical use to real-time modern systems, especially those with hard timing constraints. In this work, we integrate the probabilistic model of information propagation with a timing analysis technique to estimate a probabilistic worst case convergence time (pWCCT) for information propagation.

## Information from an Observation to a Conviction

The communication among individuals that share feelings of uncertainty and are prone to share their state and the state of the environment around them to their neighbors have been observed to be at the root of the emergence of collective behaviors^2^. The time it takes for a behavior to form and spread in a group relies heavily on the efficiency of the communication medium as well as the affinity of the individuals to receive and share information; in other words, their affinity to cooperation. The intuition here is to model the direct interaction between an individual and its neighbors and the indirect interaction with the rest of the group in a probabilistic manner to project this affinity and information loss.

**Figure 1:**
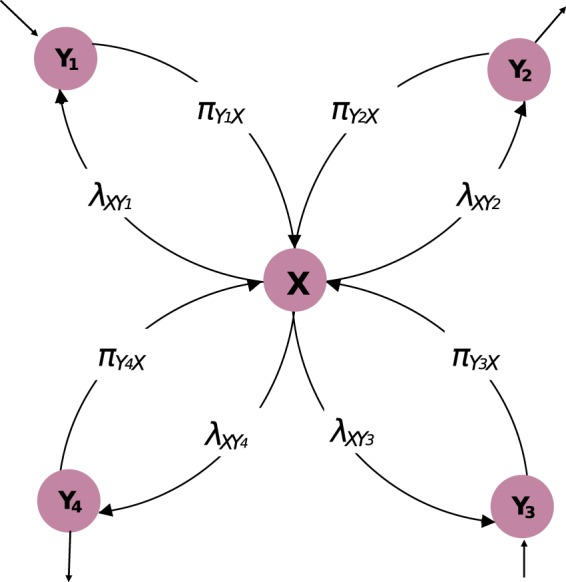
Information propagation from and to *X* through passing messages of type *λ* between *X* and its neighbors ${Y}_{i}\in {\mathscr{N}}(X)$ and *π* between ${\mathscr{N}}(X)$ and *X*.

We want to define the probability of an individual to receive information if broadcast by a different individual in the network, and not necessarily by its neighbors. Suppose that individual *A* sends an information that will reach individual *B* with probability *p*_*ab*_ = *Pr*(*B*|*A*). We define the *information conviction* as the probability of an individual to hold the information broadcast by another individual in the network. The conviction of *B* to have the information is *Pr*(*A*) · *p*_*ab*_. This is true provided *B* has a single source of information, *A* in this case. For such, we need to define what it means to share information, and how to condition the information propagation on other sources of information. The example in Figure 1 shows that *X* has multiple of these sources. We model this as a message passing system in which a message transmits the confidence of a node to pass on the information to a particular neighbor. It can be viewed as an individual sending a message to broadcast their ability to propagate the information; in other words, their conviction of holding the information and the confidence of which they are to send it to a neighbor given what its other neighbors are saying through the received messages. Hence, we recognize two types of messages:

- *Received messages* represent the messages *π* received by a node. They indicate the opinion of the source node of how likely the information is to reach a certain node from its neighboring nodes;
- *Shared messages* define the messages *λ* that a node share with its neighbors. Message ${\lambda }_{X{Y}_{1}}$ for instance describes the likelihood of *Y*_1_ getting the information from a specific source *X* and no other.

The algorithm is local in that a node relies only on the opinion of its neighbors from the received messages to build its conviction of an information reaching it given that the information was observed and broadcast from remote parts of the network. That being said, the interaction graph is defined as a directed graph that can contain cycles (See *Methods*). Initially, the only observations are the messages shared by the broadcasting source whereas all other messages are initialized to zero. To reach the information conviction of every individual considering direct and indirect interactions, messages are updated iteratively from previous observations of the information propagation in the network. We direct the reader to the *Methods* section for a detailed description of this process.

**Figure 2:**
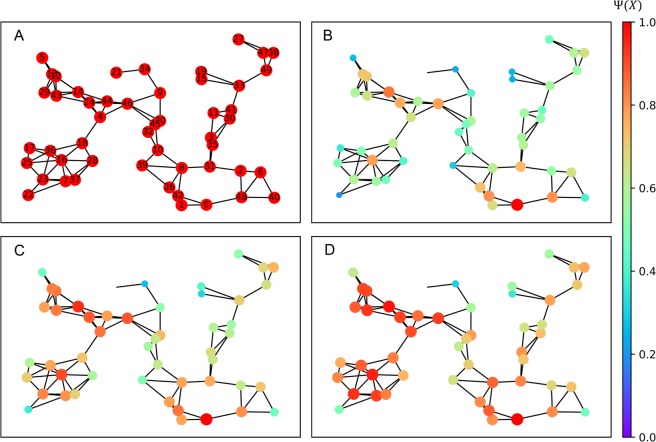
The accumulation of information conviction for the network in (**A**) for iterations (**B**) *t* = 1 (**C**) *t* = 3 (**D**) *t* = 6. We can see that as we observe new states, the conviction of certain nodes grow larger than others. This is observed to not be entirely dependent on the distance to the broadcasting source where some nodes in different regions of the network exhibit higher conviction than immediate neighbours.

For illustrative purposes, a homogeneous network of 50 individuals is depicted in Figure 2A in which the information has been set to have equal chances of reaching a neighboring node or getting lost in the process from any node in the network. Iterating over the procedure described by Algorithm 1 (See *Methods*) produces the results in Figure 2 in which we can observe the accumulation of the belief of the reception of an information broadcast at node *ν*_0_ throughout different iterations. Once the algorithm converges, the conviction of every individual to hold the information is as exhibited in Figure 2D. The neighbours of the broadcasting source are observed to have a high conviction that they hold the information, which is expected. Surprisingly though, we notice that individuals such as {*ν*_14_, *ν*_16_, *ν*_23_} far from the source and with a degree of separation higher than two nodes, in different clusters altogether for that matter, have quite a high information conviction. This tells us that the propagation of information in a network of interacting individuals might not necessarily depend entirely on the distance to the source of the information and line of sight. This leads us to question whether centrality is the reason behind these observations. This notion is further studied in later sections where we show its validity and determine the circumstances under which it no longer holds.

## Probabilistic Worst Case Convergence Time

The transfer of knowledge acquired from studying animal group behaviors to artificial systems has seen a surge of interest in the research community as of late. In light of this, there is a need to study certain characteristics that are inherent to these modern systems; which might, in turn, aid in understanding some behaviors in animals and humans alike that are still unpredictable. One of these characteristics is *timing performance*. In particular, this paper aims at providing a probabilistic estimate of the worst case time for a group to reach a consensus over a piece of information. We develop a methodology to obtain the exceedance probability curve (or Complementary Cumulative Distribution Function) which describes the probability that the convergence time will exceed a certain threshold. We refer to this as the probabilistic worst-case convergence time (pWCCT). This is of a particular interest to a system designer whom might have strict timing restrictions: for example, the need to ensure that the time for a robot swarm to autonomously agree on a task assignment doesn’t exceed a certain deadline.

For every individual *ν*_*i*_, we define ${I}_{{\nu }_{i}}(t)$ as a probability mass distribution (PMD) which describes the probability of the individual *ν*_*i*_ to receive the information from its neighbors at time *t*. The intuition is that, for an individual to receive the information at time *t* = *T*, it suggests that its neighbors that hold the information have failed to transfer it at *t* < *T*. The PMDs ${I}_{{\nu }_{i}}$ represent the timing behaviour of corresponding individuals *ν*_*i*_. To estimate the timing for a consensus to be reached, we need to look at the timing behavior of the network. Two elements are to be considered: (i) how to translate the individual timing behaviors into a group timing, namely a probabilistic worst case convergence time (pWCCT); and (ii) what possible structures to study to achieve a bound on the pWCCT.

**Figure 3:**
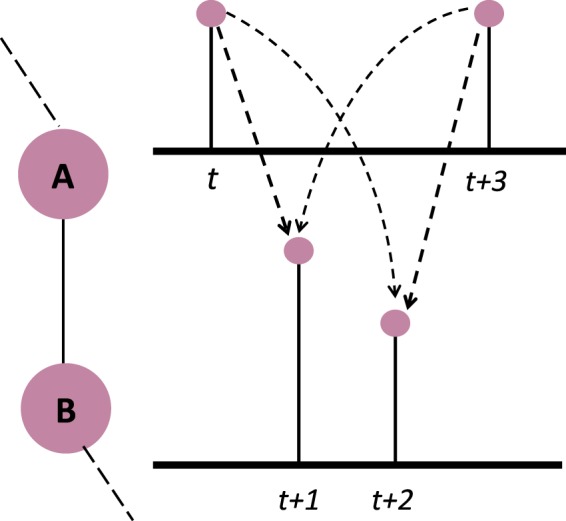
*PMD*s of *A* and *B* in which the arrows represent every possible time when both *A* and *B* have the information. The arrow linking times *t* and *t* + 1 defines the case in which A receives the information at time *t* but fails to propagate to B which receives it at time *t* + 1 instead.

In Figure 3, information is propagated to *A* and *B* with their corresponding PMDs. To determine the timing of this information flow, we need to enumerate the times at which both *A* and *B* have the information, and define the probability to reach every state. In the figure, every arrow represents one of these cases. Convolving the two distributions turns out to produce a probability distribution that describes these possible cases. To estimate the exact timing of the information propagation, one needs to consider all possible paths the information might take to propagate from an individual to the rest of the network. The complexity of this grows exponentially as the size of the network increases. However, since our main concern lays in estimating the worst case convergence time, it is sufficient to consider the longest path in the graph of the network. This is similar to picking one node at a time to receive the information from the rest of the network at every time-step. As one can expect, this consideration results in an upper bound of convergence time that is quite pessimistic. In reality, individuals in a group are more likely to transmit information to multiple individuals at the same time. In order to tighten this estimation of the probabilistic worst convergence time, instead of considering the longest path that spans all nodes, we look for a spanning tree with minimum branching. Among other assumptions (detailed in *Methods*), we have to assume that the information propagates sequentially to all the individuals, as the inclusion of branches hinders the use of convolution. The way to combine the PMDs of the nodes in the branches with the rest of the spanning tree is by defining a merge operator (described more thoroughly in *Methods*). This takes into account the possibility that certain individuals receive the information at the same time, akin to a parallel process.

## Results

As has been mentioned previously, collective behavior is the product of sharing some kind of information among a group of individuals. Our work spans any system that can be represented as a network of agents propagating an information. In what follows, we illustrate the efficiency of our framework in a few examples of information propagation, namely rumour spreading and consensus reaching in a robot swarm. In this section, we showcase the model presented in this paper as (1) a way to study behaviors and understand the reasons certain events progress in the manner that they do; and (2) as a practical solution to issues encountered in engineered collective systems.

### A Rumour and its Counter-Rumour

**Figure 4:**
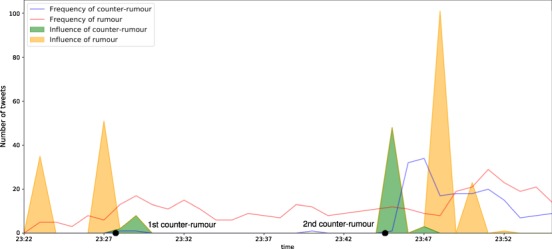
Progression of the rumour and the counter-rumours in terms of time which reveals two main attempts at correcting the false claim with differing reactions.

We study the case of rumour spreading as an example of information propagation in social media. We analysed 7 major news events from the PHEME rumour dataset^32^ that spans 297 twitter conversations discussing rumours. We focus on one of the rumours that spread during the Sydney hostage situation of 2014. Figure 4 shows the progression of the rumour that claims that hostages were held by terrorists associated with ISIS. The rumour started by the following tweet:

@User_zero: SYDNEY SIEGE: Gunman forces hostages to hold up ISIS flag in window. [2014-12-14 23:27:39]

We observe the tweets that counter-attacked the false rumour and their spreading throughout the network. We notice an explosion of tweets denying the rumour at around 23:45 after a tweet published by a user that we refer to as Bob (For confidentiality purposes):

@Bob: Flag in window of Sydney Lindt cafe not an ISIS flag. Reads: ‘There is no God but Allah and Muhammad is the messenger of God’. [2014-12-14 23:45:51]

However, this was not the first attempt at correcting the false rumour. A tweet previously published by a different user (Alice) revised the claim with visual evidence:

@Alice: These not the same. 1st Shahadah flag, 2nd is specifically claimed by IS(ISIS). [2014-12-14 23:29:26]

In an endeavour to reach an understanding as to why the first counter-tweet by Alice didn’t have much of an impact on correcting the rumour whereas the tweet by Bob did, we study the spreading of the information as modelled in previous sections and examine interesting patterns. The procedure to build a group interaction model is fully described in the *Methods* Section, which is then used to model the information propagation for two different scenarios to obtain the conviction of every user in the network to have the information. The first scenario represents the propagation of the information in the network from Alice and the second scenario sees the information spreading from Bob.

**Figure 5:**
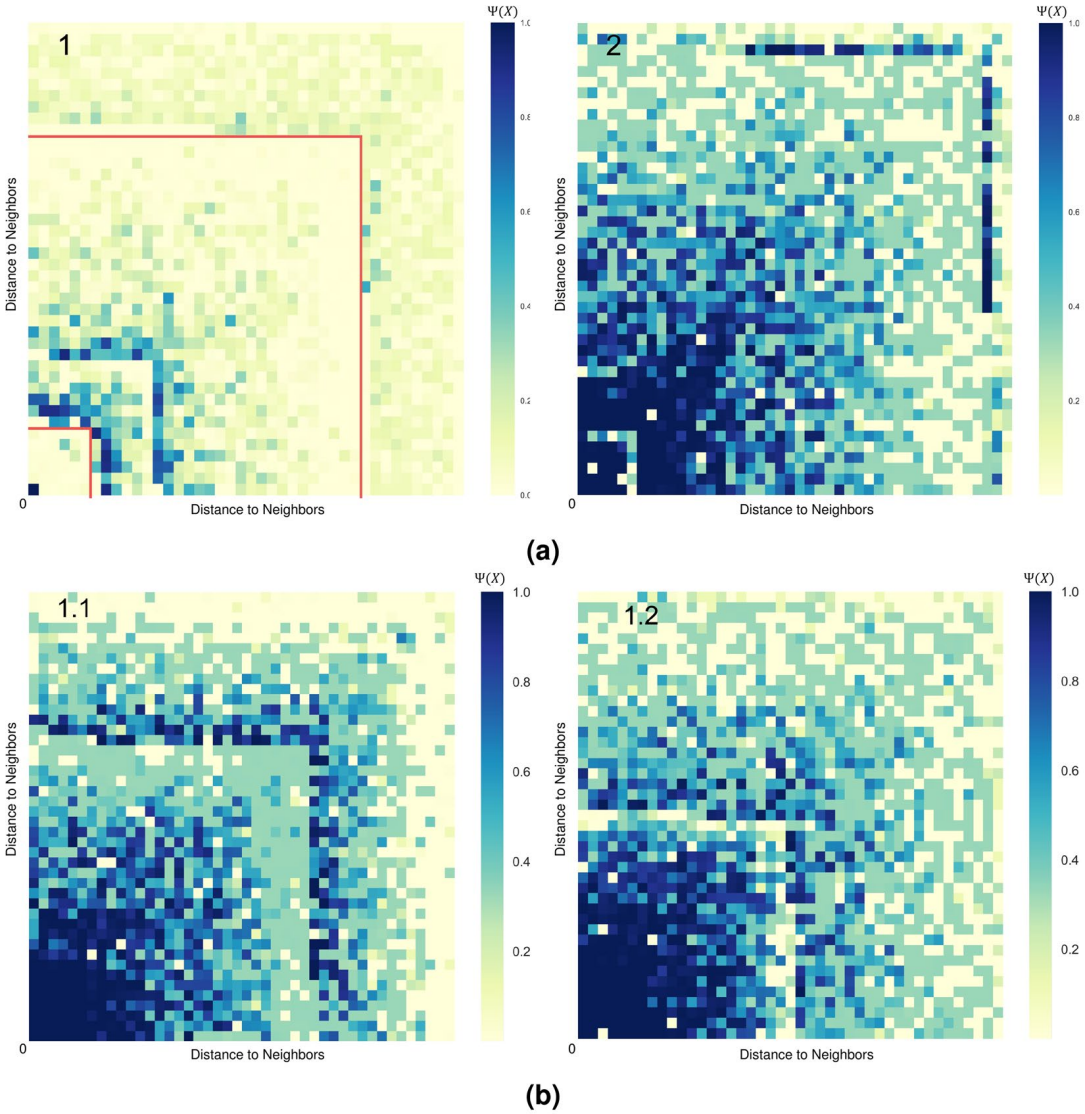
(**a**) Heat maps illustrating the probability of the information spreading from (1) Alice and (2) Bob starting at the bottom-left corners to their neighbors. (**b**) Heat maps for critical users in the intermediate neighborhood of User Alice that exhibit higher probabilities of information propagation than Alice.

Figure 5a illustrates the conviction of receiving the information by every individual in the network if the information was propagated initially by Alice and Bob respectively, based on their distance from the source. The heat map generated for Bob shows a wider outreach and propagation of the information to the other users in the network compared to Alice. This in part explains the reason behind the progression of information observed in Figure 4. We also explore how this analysis could be exploited to prevent rumour spread. Figure 5a.1 can be divided in three sections: the first is Alice propagating the information but failing to reach its immediate followers; the second section can be observed to have a sudden darkening area which indicates a user with a great conviction of receiving the information from Alice and a higher influence on their immediate followers and indirect relationships; and a last section, two-thirds through the network, that encounters another user able to further propagate the information through the rest of the network.

Analyzing the spreading of information starting at these critical users results in the heat-maps of Figure 5b. Both maps demonstrate a wider outreach then that observed for Alice. These users exhibit high conviction as well as high influence on their direct and indirect neighbours. This could be an incentive to rely on the framework to detect critical users that will spread the right information in times of crisis and quickly quench any rumours that might arise unforeseen chaotic behaviors.

### Timing and Resilience

We experimented with our framework as an analysis tool where we estimate timing characteristics and study the resilience of a network to loss of information.

#### pWCCT and Kilobots

An interesting question to answer is how fast the spreading of infection could occur; either through the body, similar to cancerous cells contaminating neighbouring healthy cells^33^ or at the population level, such as the spread of influenza. A recent work tackled this issue to determine the takeover time. The propagation model by Ottino *et al*.^34^ assumed an infected node transmits the infection to a single neighbor at random every time-step from any infected individual in a network. With our framework, we go beyond this simple model: whereas we don’t claim to estimate the exact probabilistic distribution of the time for infection spreading, we model the probability of the infection spreading from any infected individual and upper bound the worst takeover time in a probabilistic manner under two assumption: (a) the infection can spread to more than one individual in a single time-step (b) as well as consider that every individual might have a different transmission affinity.

**Figure 6:**
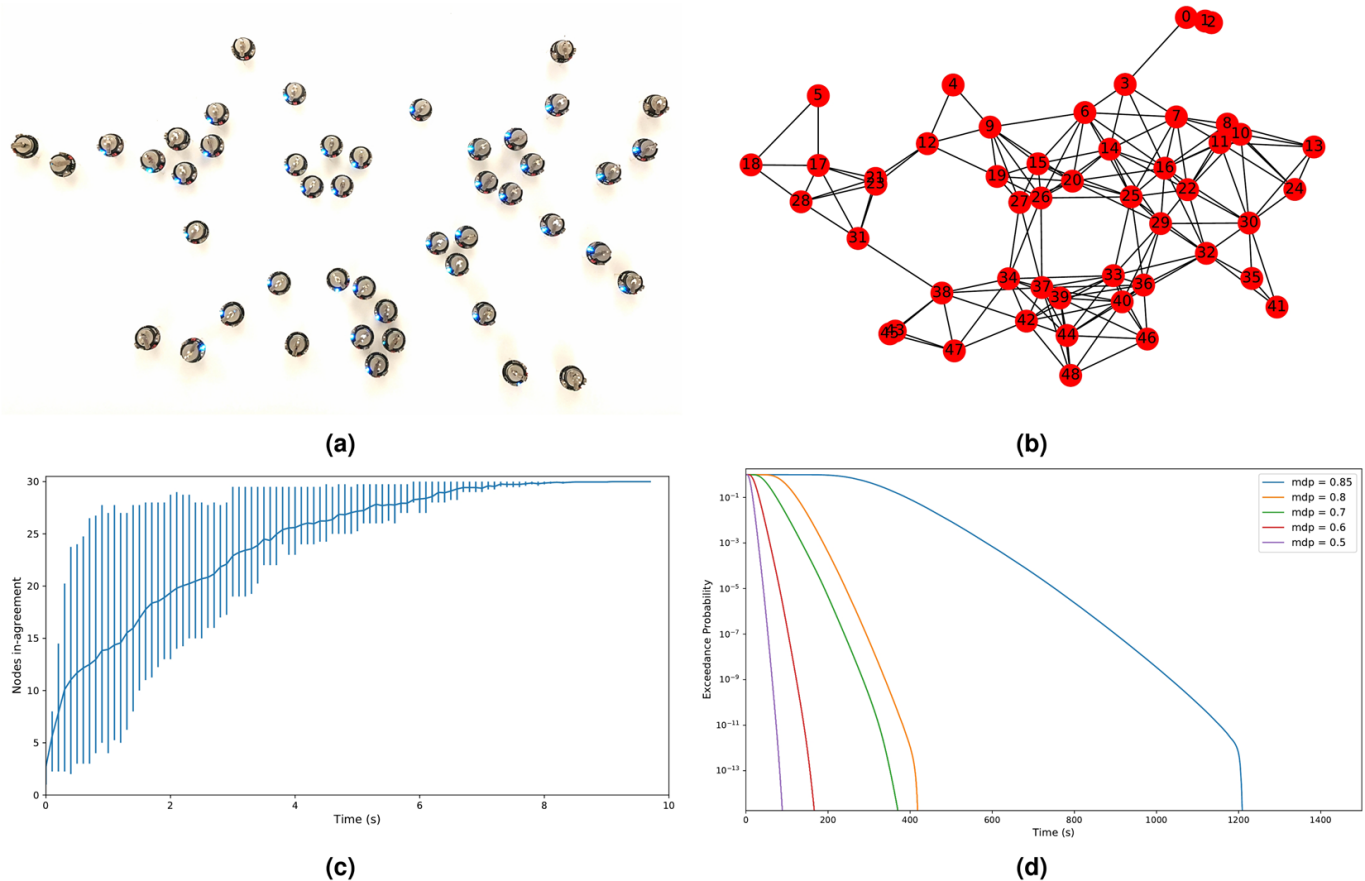
For the scale-free topology in (**a**), we show the interaction graph in (**b**), the time for the swarm of Kilobots to reach complete agreement (**c**) and its corresponding estimation of pWCCT (**d**).

In here, we employed a swarm of small robots to emulate the propagation of information–infection in the example above–throughout a network. To validate the ability of our model to upper bound the worst case convergence time towards a consensus, a set of experiments on a real swarm of robots was performed in which a number of Kilobots^35^ form a swarm of different topologies (Figure 6a shows the robots in a scale-free topology) and the aim is to study the time to converge to a consensus over a piece of information. Kilobots are simple robots that rely on infra-red communication which renders message transmissions very susceptible to noisy environments. Our motivation to experiment on a real robot swarm is to appraise the performance of our framework when it deals with physical characteristics of the swarm communication such as collision, interference, etc. that are difficult to simulate.

Figure 6c plots the results obtained for 30 runs of the same experiment on a scale-free topology. We observe that the distribution of the convergence times has a high variance and that is partially due to the fact that the scale-free topology contains a number of clusters connected by a small number of links. This renders the nodes at these links highly critical and whether they succeed to transmit the message or not highly affects the convergence time. The convergence time recorded for this particular topology was in the range [3.8, 17.6](*s*).

Looking at what we estimated as the worst case convergence time expected for this topology, we examine Figure 6d. We plot the exceedance probability distributions for *mdp* ∈ [0.5, 0.6, 0.7, 0.8, 0.85] varying the message drop probability (*mdp*). Our interest lays in the plots for *mdp*_1_ = 0.6 and *mdp*_2_ = 0.7 (Refer to *Methods*) which show the worst convergence time estimated at different exceedance probabilities. In other words, *WCCT* = 170(*s*) and *WCCT* = 360(*s*) for *mdp*_1_ and *mdp*_2_ respectively, exceeded with a very low probability of *Pr* = 10*e*^−13^. This upper bounds the time to convergence for the swarm of Kilobots, including extreme cases. The pWCCT estimation based on both convolution and merge presented here defines a *safe* upper-bound to the information propagation in a network that might be pessimistic when only ordinary situations are expected. However, its use is promoted in hard real-time systems that call for strict requirements on their timing behaviors such as robot-aided space exploration or robot emergency responders. This also offers a flexible measure to upper bound this timing characteristic by varying the exceedance probability threshold.

The advantage of our model over what is proposed in literature is its ability to estimate a pWCCT for different topologies, and to be adaptable to different scenarios. In addition to the scale-free topology we presented here, results on other topologies such as a snake-like topology, and a topology where the robots are randomly distributed with obstacles can be observed and are presented later as additional results.

#### A Chain is No Stronger than Its Weakest Link

From our study of rumour propagation, it is clear that preventive measures that rely on robustness analysis of the network are of utmost importance. This extends to non-biological systems, such as wireless sensor networks (WSNs). Designing a network that is entirely fault-tolerant can be too expensive, in terms of time, cost, and expertise. Our model can be a smart solution to select critical nodes in wireless sensor networks (WSNs) to be hardened against faults. Broadly speaking, our model is a tool to analyze a network and detect the weaker individuals that interfere with the propagation of information.

**Figure 7:**
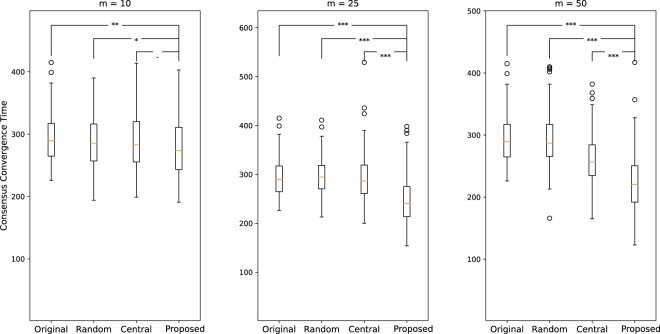
pWCCT for a random geometric network in its original form, and different section methods as the number of selected elements *m* is varied to (**a**) *m* = 10, (**b**) *m* = 25, and (**c**) *m* = 50.

To demonstrate this, we simulate a robot swarm in a random geometric topology in which a selected number of robots have been hardened, i.e. they have been given message drop probabilities *mdp* = 0 to ensure message transfer. In addition, instead of working with a homogeneous swarm, we randomly assign different *mdp* for every node in the graph. The purpose behind this choice is to observe networks that are heterogeneous in terms of information propagation (e.g. due to individual preferences, different noise levels in the environment, etc.). We compare against random selection, in which a set of nodes are picked at random. We also mentioned previously that our observations of information propagation might be related to centrality. We study these claims by comparing our method against a centrality-based selection which relies on the topology of the network and picks the nodes that have a high out-degree. We run every scenario 100 times, while we vary the number of selected elements *m*. This produces the results summarized in Figure 7.

While there is no major improvement in terms of shorter convergence time when the number of selected elements is small, we observe a major decrease in the time to reach consensus with *m* = 50 selected elements. The first observation shows that although our method reached global consensus in less time than the original scenario (with no hardened elements), the times reached were statistically similar to a centrality-based approach. In this case, since *m* is small, both techniques were choosing geometrically similar nodes.

The choice of working with a heterogeneous swarm, at least in terms of *mdp*, is highlighted in the results of Figure 7b,c. The idea is that a centrality-based technique selects nodes with high connectivity regardless of their information conviction ${I}_{{v}_{i}}$. Instead, we locate the weaker links in the network that might hinder the spreading of information throughout the swarm, especially in terms of convergence time. We do so by identifying the nodes that have a high conviction of obtaining the information, but are failing to transmit the information to their neighbours. In other words, we favor a node that we consider a weak link based on its ability to further propagate the information in the network, even if its connectivity is low. For the sake of brevity, we did not include the results for a *homogeneous* network, but it is interesting to note that our model exhibits similar behavior as centrality-based methods when considering a homogeneous swarm of robots where the *mdp* is constant across the network. This leads us to conclude that centrality is a good measure to select influential nodes. However, by itself, it fails to promote the propagation of information especially in more complex configurations.

## Discussion

Information propagation effects have been categorized by Arif *et al*.^14^, in the case of rumours, into 4 patterns: Giant, Snowball, Fizzle and Babble. The giant and snowball effects, which both exhibit high derivative information propagation by high and low exposure individuals respectively, were of a particular interest since they present patterns that emergency responders look out for to maximize spreadability and stave off the emergence of chaotic behaviors. We observe both of these effects in Figure 5 from Bob and Alice respectively. Although the giant effect could be intuitively interpreted, the snowball effect is an observed fact that is not completely understood. In here, we introduced a simple but sufficient model of information propagation for the aim of studying emergence of behaviors. The purpose here was to explore the effect of stripping the propagation model from scenario-specific details, such as the volume of shared tweets or the rate at which the information is transmitted, etc. This showed that the few assumptions taken in modeling information propagation were enough to discern the patterns that lead to the emergence of the observed event. The study promotes the practice of bottom-up investigation when it comes to modeling information flow and to properly identify and isolate the originator of specific events and behaviors; as is the case with giant and snowball effects in rumour propagation research. For instance, we show that, although the snowball effect starts with low exposure individuals, it is mainly due to highly influential individuals picking up the information from low exposure sources. Since the model quantifies the information conviction of individuals, it is able to detect easily-influenced individuals which are themselves apt to influence other individuals. Being able to identify the major players in information propagation is of utmost importance especially in crisis situations. We suggest that the framework can readily be used to spread the right information and maximize the likelihood of giant effects. In fact, we exploited this characteristic to analyze artificial networks in which the framework was used to detect the weaker links that hinder information spreading which, as a result, produced more resilient systems. The framework was extended with a timing analysis technique to study information consensus in a group. We showed that despite the few assumptions taken to model the information propagation, which neither fully express the intricacies of the diffusion of information nor the environment, it is able to provide a probabilistic measure of the worst convergence time towards a consensus. Future work could see the framework extended to uncover the history of current events, such as determining the time and place for the origin of an infection. Admittedly, the framework presented could model a single information with multiple sources. However, future studies could see an extension of the framework to handle multiple, possibly conflicting, information in the network.

## Methods

### Group interaction model

The interaction between the group individuals is represented by a directed graph $G=({\mathscr{V}},E)$ in which the vertices ${\mathscr{V}}=\{{\nu }_{i}|i\in (0,n]\}$ depicts the *n* individuals in a group and *E* = {*e*_*ij*_|(*i*, *j*) ∈ (0, *n*]} the information flow from *ν*_*i*_ to *ν*_*j*_. The graph can have cyclic interactions where the individuals exchange information in both directions, which makes the existence of edges *e*_*ij*_ and *e*_*ji*_ possible. This is observed in a pod of bottlenose dolphins where whistles are exchanged to identify whether a dolphin belongs to a certain group^36^. Since we want to study a group of individuals in noisy environments that are apt to not cooperate, a probability of the information propagating from *ν*_*i*_ to *ν*_*j*_ is defined as *p*_*ij*_ = *Pr*(*ν*_*j*_|*ν*_*j*_) and is assigned to every pair (*ν*_*i*_, *ν*_*j*_).

### Conviction through message passing

The message-passing framework implements the idea that a node builds its conviction on an information reaching it, given the information was observed at one or multiple sources, by “listening” to the opinion of its neighbors about their own observations. This happens through an exchange of messages loosely based on the message passing algorithm described in^37^. In other words, the messages are a way to virtually strengthen a node’s belief that it will hold a piece of information. This is done by observing the likelihood of its neighborhood to bring the information to it. The observations are modelled through the received messages. The node, by updating its conviction, shares a message to broadcast its conviction to hold and transmit the information. The existence of cycles in the graph imposes a recursive process for a node to iteratively reinforce its opinion until all messages converge.

The example in Figure 1 shows that *X* has multiple neighbors in which we recognize two types of messages; *Received messages* and *Shared messages*. The received messages are built on the direct relationship between a node *X* and its neighbors ${\mathscr{N}}(X)$ and the indirect influence from its second degree neighbors ${\mathscr{N}}({\mathscr{N}}(X))$. They are interpreted as a node receiving the opinion of its neighbors on the likelihood that they will transmit the information given the state of the rest of the network and are defined as:

1 $${\pi }_{{Y}_{i}X}^{(k+1)}=\frac{1}{|{\mathscr{N}}({Y}_{i})|}Pr(X|{Y}_{i})\sum _{Z\in {\mathscr{N}}({Y}_{i})}\,{\lambda }_{Z{Y}_{i}}^{(k)}$$

**Algorithm 1:**
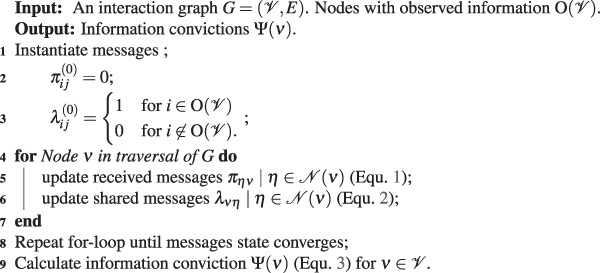
Building conviction through message passing.

Shared messages are of a particular interest since they can be decomposed to represent how much influence a node has on its individual neighbors. Message ${\lambda }_{X{Y}_{1}}$ for instance describes the level of conviction that *Y*_1_ will get the information from a specific source *X* and no other. They are defined as:

2 $${\lambda }_{X{Y}_{i}}^{(k+\mathrm{1)}}=\frac{1}{|{\mathscr{N}}(X)|-1}\sum _{{Y}_{j}\in {\mathscr{N}}(X)j\ne i}\,{\pi }_{{Y}_{j}X}^{(k)}\,Pr({Y}_{i}|X)$$

The summation excludes the message ${\pi }_{{Y}_{i}X}$ received from *Y*_*i*_ since the shared message ${\lambda }_{X{Y}_{i}}$ represents the degree to which *Y*_*i*_ is convinced that the information is coming from *X*, which eliminates the possibility that ${\pi }_{{Y}_{i}X}$ will be holding the information.

We define Ψ(*X*) as the conviction of individual *X* that it holds the information observed to have spread from a particular node in the network and is defined as

3 $${\rm{\Psi }}(X)=\frac{1}{|{\mathscr{N}}(X)|}\sum _{{Y}_{i}\in {\mathscr{N}}(X)}\,{\pi }_{{Y}_{i}X}$$

Given the interaction graph defined above and an observation of the information at one or multiple broadcasting sources, the way to properly build the conviction of the other individuals in the network is by traversing the graph and gradually updating the state of the messages as information is observed. We do this in an iterative process that is summarized in Algorithm 1. Intuitively speaking, since the information is only observed at the sources, the messages are initialized to zero since they depict the conviction of an individual having the information, except for the messages shared by the broadcasting nodes. The state of the messages is then updated as dictated by Equations 1 and 2 by following the flow of the information and repeating the process until convergence. We are able then to define an information conviction for every node in the graph.

### Probabilistic worst case convergence time

The multiple outbreaks of Spruce Budworms that ravaged north-American forests almost every decade of the first half of the 20th century^38^ is a scenario of infection propagation that is still being studied extensively. Having a tool to estimate the time for an infection to spread in a community could lead to better prevention methods and open doors to understanding the propagation patterns. The probabilistic model defined so far is an essential part to reach this goal.

For every individual *ν*_*i*_, we define a probability mass distribution (PMD) ${I}_{{\nu }_{i}}(t)$ that describes the probability of the individual to receive the information from its neighbors at time *t*.

4 $${I}_{{\nu }_{i}}(t)=p{(1-p)}^{t}$$

where $p={{\rm{\Psi }}}_{{\nu }_{i}}$ represents the conviction that individual *ν*_*i*_ will receive the information from one of its neighbors.

The PMDs ${I}_{{\nu }_{i}}$ represent the timing behavior of corresponding individuals *ν*_*i*_. To estimate a bound on the timing for a consensus to be reached, we need to look at the collective timing behavior in the form of a probabilistic worst case convergence time, which rises two concerns: (i) how to translate the individual timing behavior into a group timing and (ii) what possible structures to study in order to achieve a tighter bound on the pWCCT.

Looking at the first issue, we look at how to combine all possible ways in which the information could propagate in the network in terms of timing. We rely on the convolution operator to implement this;

5 $${I}_{{\nu }_{i}}\,\ast \,{I}_{{\nu }_{j}}(t)=\sum _{s=-\,\infty }^{\infty }\,{I}_{{\nu }_{i}}(s){I}_{{\nu }_{j}}(t-s)$$

Since the interest lays in estimating the worst time to convergence, and in order to estimate a tighter upper-bound on the pWCCT, instead of considering the longest path that spans all nodes, we look for a spanning tree with minimum branching.

**Figure 8:**
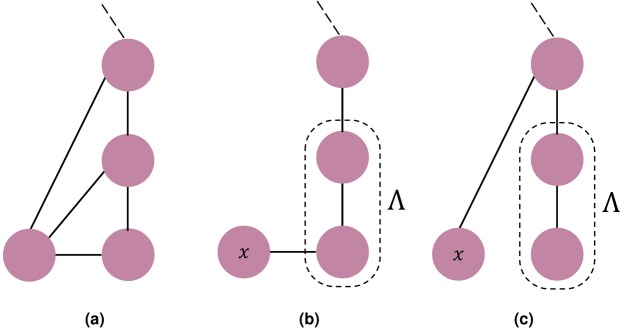
Example graph to demonstrate (**b**) sequential propagation of information from Λ to *x* that requires convolution of PMDs and (**c**) the less pessimistic structure of seeing *x* as part of a branch, akin to parallel process, which requires the merge operator.

For the convolution to work however, we have to assume that (i) the PMDs are independent and identically distributed (i.d.d) and (ii) the information propagates in a sequential manner to all the individuals (See Figure 8). The inclusion of branches in the considered structure hinders the use of convolution solely. The way we go about combining the nodes in the branches with the main spanning tree is by defining a merge operator:

6 $${I}_{{\nu }_{i}}\uplus {I}_{{\nu }_{j}}(t)={I}_{{\nu }_{j}}(t)\sum _{s\le t}\,{I}_{{\nu }_{i}}(s)+{I}_{{\nu }_{i}}(t)\sum _{s < t}\,{I}_{{\nu }_{j}}(s)$$

which looks into the probability of the information taking longer to reach the nodes in the branch than the main path and vice-versa. This takes into account the possibility that certain individuals receive the information at the same time, akin to a parallel process. In the structure of Figure 8c, the merging will look at the probability of the information reaching *x* at time *t* while the information has already spread to Λ at time *s* ≤ *t* and vice-versa. This reduces the pessimism of the convolution-based pWCCT estimation and offers a tighter bound.

#### **Theorem 1.**

*Let* Λ *be a simple path and x be a node in a graph G such that* $x\notin {\rm{\Lambda }}$ *then*, *merging I*_*x*_(*t*) *and I*_Λ_(*t*) *yields a tighter bound on the pWCCT than convolution*.

We introduce a small lemma to prove this theorem.

#### **Lemma.**

*Given a double summation*, *interchanging the order yields*,

$$\sum _{j=0}^{n}\,\sum _{i=1}^{j}\,f(i,j)=\sum _{i=0}^{n}\,\sum _{j=i+1}^{n}\,f(i,j)$$

*This can be simply reached by looking at the double summation as one sum* ${\sum }_{t\in \{(i,j)|j\le i\le n\}}\,f(t)$.

#### *Proof*.

Since the pWCCT is an exceedance probability distribution, to prove tightness of bound, it is enough to show that the CDF of merging at any given time *t* is larger than that of convolution. Formally, we want to prove that

$$\sum _{k=0}^{t}\,{I}_{{\rm{\Lambda }}}\uplus {I}_{x}(k)\ge \sum _{k=0}^{t}\,{I}_{{\rm{\Lambda }}}\,\ast \,{I}_{x}(k)$$

The convolution of two PMDs can be rewritten as a *Cauchy Product*,

$$\sum _{k=0}^{t}\,{I}_{{\rm{\Lambda }}}\,\ast \,{I}_{x}(k)=\sum _{k=0}^{t}\,{I}_{x}(k)\sum _{s=0}^{t}\,{I}_{{\rm{\Lambda }}}(s)$$

For the merge operator, given Equation 6, we study two cases;

For *k* ≥ *t*:

$$\begin{array}{rcl}\sum _{k=0}^{t}\,{I}_{{\rm{\Lambda }}}\uplus {I}_{x}(k) & = & \sum _{k=0}^{t}\,({I}_{{\rm{\Lambda }}}(k)\sum _{s=0}^{k}\,{I}_{x}(s)+{I}_{x}(k)\sum _{s=0}^{t}\,{I}_{{\rm{\Lambda }}}(s)+{I}_{x}(k)\sum _{s=t+1}^{k}\,{I}_{{\rm{\Lambda }}}(s))\\  &  >  & \sum _{k=0}^{t}{I}_{{\rm{\Lambda }}}\,\ast \,{I}_{x}(k)\end{array}$$

For *k* < *t*:

$$\sum _{k=0}^{t}\,{I}_{{\rm{\Lambda }}}\uplus {I}_{x}(k)=\sum _{k=0}^{t}\,({I}_{{\rm{\Lambda }}}(k)\sum _{s=0}^{k}\,{I}_{x}(s)+{I}_{x}(k)\sum _{s=0}^{t}\,{I}_{{\rm{\Lambda }}}(s)-{I}_{x}(k)\sum _{s=k+1}^{t}\,{I}_{{\rm{\Lambda }}}(s))$$

Given the lemma, the last term can be rewritten as:

$$\begin{array}{rcl}\sum _{k=0}^{t}\,{I}_{{\rm{\Lambda }}}\uplus {I}_{x}(k) & = & \sum _{k=0}^{t}\,({I}_{{\rm{\Lambda }}}(k)\sum _{s=0}^{k}\,{I}_{x}(s)+{I}_{x}(k)\sum _{s=0}^{t}\,{I}_{{\rm{\Lambda }}}(s)-{I}_{{\rm{\Lambda }}}(k)\sum _{k=1}^{t}\,{I}_{x}(s))\\  & = & \sum _{k=0}^{t}\,({I}_{{\rm{\Lambda }}}(k){I}_{x}\mathrm{(0)}+{I}_{x}(k)\sum _{s=0}^{t}\,{I}_{{\rm{\Lambda }}}(s))\\  & \ge  & \sum _{k=0}^{t}\,{I}_{{\rm{\Lambda }}}\,\ast \,{I}_{x}(k)\end{array}$$

### Rumour data collection

We relied on the Twitter API to collect the relevant information to build the network connecting users Alice and Bob to the users involved in the rumour and compile the data in the form of a group interaction model as described above. The parameter of most importance in the graph model is the probability of an individual transmitting an information to its neighbors *p*_*ij*_. In the context of social media networks and interactions among humans, this refers to what is commonly addressed as *social influence*. On that account, metadata were extracted to represent the influence probability *p*_*ij*_ defined as the probability of user *i* to influence the opinion of user *j*. It is common in literature to rely on the rate of communication to quantify the influence^39,40^. In here, we define this parameter as:

$${f}_{c}(i\mapsto j)=\frac{{\rm{Number}}\,{\rm{of}}\,{\rm{tweets}}\,i\mapsto j}{{\rm{Last}}\,{\rm{1000}}\,{\rm{tweets}}}$$

In order to better represent the influence between individuals, we estimate the influence based on two other quantities; the level of trust between users *f*_*t*_ and the popularity of the user in the network *f*_*p*_.

$${f}_{t}(i\mapsto j)=(\begin{array}{ll}\mathrm{True} & (i,j)\in {\rm{\Phi }}(j)\cup {\rm{\Phi }}(i)\\ \mathrm{False} & otherwise\end{array}$$

where Φ(*i*) represents the set of individuals following user *i*.

$${f}_{p}(i)=\frac{|{\rm{\Phi }}(i)|}{\mathop{{\rm{\max }}}\limits_{k\in {\mathscr{V}}}|{\rm{\Phi }}(k)|}$$

We then define the probability of *i* influencing *j* as a weighted combination of these quantities:

7 $${p}_{ij}={\omega }_{c}\,{f}_{c}+{\omega }_{p}\,{f}_{p}+{\omega }_{t}\,{f}_{t}$$

where $${\sum }_{k}\,{\omega }_{k}=1$$.

We explored different values to the weights and we noted that giving a high weight to the rate of communication *f*_*c*_, following the common trend in literature, didn’t show any discernible patters. We observed the same result when giving a high weight to the trust factor *f*_*t*_. The popularity factor, on the other hand, with a slightly higher weight, resulted in the patterns observed in Figure 5. This is highly pertinent to the fact that the case we study is the propagation of a rumour in a wide-scale event, such as a siege, in which the popularity of the propagator plays a bigger role in influencing their audience. We hypothesize that a study of a smaller scale such as among family and peers would require the assignment of higher weights to the communication rate and trust factors.

### pWCCT experiments

#### Experimental Setup

The communication protocol in the swarm follows the strategy proposed by Pinciroli *et al*.^41^ labelled *Virtual Stigmergy*; which is inspired by communication among insects and is robust to sharing information in large swarms even under noisy conditions. The information is stored as a timestamped tuple (key, value) and transmitted in a message to a robot’s neighborhood. We encourage interested readers to go over the paper to fully understand how Virtual Stigmergy works. In here, we limit the text to the elements necessary to understand the experiment setup. Virtual Stigmergy states that a robot updates its tuple space only if it receives a (key, value) pair that either does not exist in its table or a pair with a higher timestamp; which indicates that the value received is more up-to-date than the stored value. In these cases, the robot will update its table and broadcast a message to its neighbors in order to share the updated information. In this manner, the information propagates from a source node to the rest of the swarm even if the network is not strongly connected.

To ensure convergence, in these experiments, aside from the broadcasting procedure described above, the robots broadcast a message containing their state every period of time *T*_*s*_. This time period models the time-step time that we mentioned in a previous section and is essential to build the PMDs (Equation 4) in which a robot fails to receive a message from its neighbors in the time *kT*_*s*_ < *t* < (*k* + 1)*T*_*s*_.

We recall that the Kilobots rely on infra-red communication which is unreliable and highly sensitive to the experiment’s environment. For such, a set of separate experiments were performed to gauge the message drop probability of the Kilobots, labelled so forth *mdp*. Although the quantity was highly sensitive to the saturation level of the communication space and the environment such as the ambient light, the reflection of the communication medium, etc., the recorded probabilities were in the range *mdp* ∈ [0.63, 0.78].

#### Additional Results

**Figure 9:**
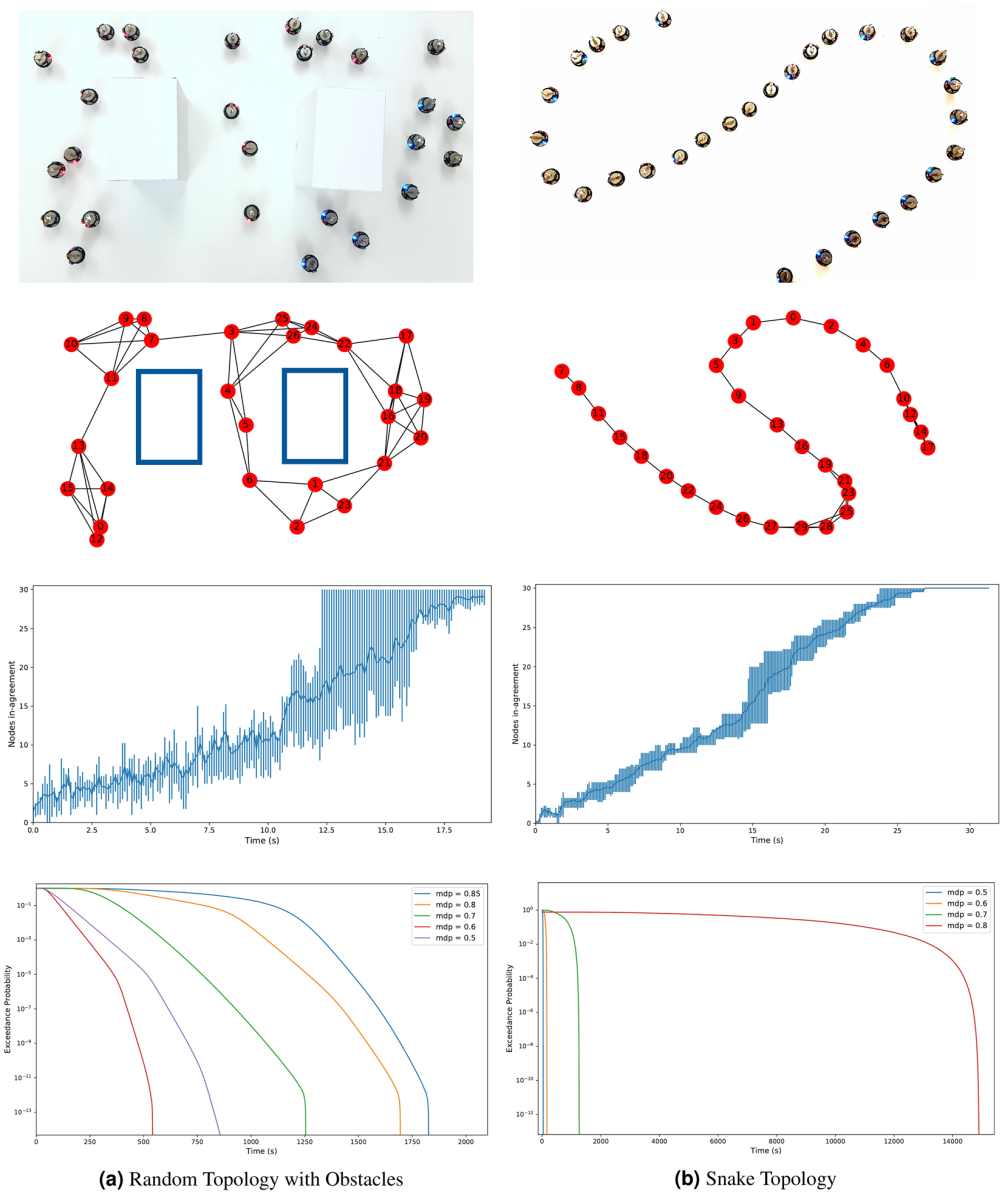
Time for a swarm of Kilobots to reach complete agreement and its corresponding estimation of pWCCT for (**a**) a random topology with obstacles and (**b**) a snake topology.

Figure 9 summarizes the results obtained for two different topologies: (a) a randomly distributed swarm with two obstacles to limit the communication between sections of the swarm and (b) a snake-like topology similar to a line topology which differs in the fact that the out-degree of every node is not forced to 1. The figures plot the times to convergence from 30 runs on Kilobots and their corresponding pWCCT estimations from our model.

The first thing that we observe is that the variance in estimations for different *mdp* differs from one topology to another which is expected since our model relies on the topology to estimate the pWCCT, more specifically, on the structure considered to combine the individual timing behaviors. Particularly, we observe that the estimations for the snake topology are more pessimistic as the *mdp* increases. This is due to fact that the structure of the spanning tree with minimum branching is closer to that of the longest path which implies that the convolution operator is mostly used. As proven before, the convolution introduces pessimism to the estimation which explains the results of Figure 9b.
